# Anti‐HIV Serological Profiles and Concordance of Chemiluminescent Immunoassay Screening With a Supplemental Rapid Test Among Blood Donors in Côte d’Ivoire

**DOI:** 10.1155/jimr/4683550

**Published:** 2026-09-03

**Authors:** M’boh Epi Reine Elisabeth N′Gou, Mamadou Yassongui Sekongo, Saydou Kabore, Nardianhio Soro, Natacha Fleur Nenelou, Liliane Siransy, Bamory Dembele

**Affiliations:** ^1^ National Blood Transfusion Center, Abidjan BP V15 Abidjan, Côte d’Ivoire; ^2^ Faculty of Medicine and Biomedical Sciences, Félix Houphouët-Boigny University (UFHB), Abidjan 01 BP V 34, Abidjan 01, Côte d’Ivoire, univ-fhb.edu.ci; ^3^ Faculty of Pharmaceutical and Biological Sciences, Félix Houphouët-Boigny University (UFHB), Abidjan 01 BP V 34, Abidjan 01, Côte d’Ivoire, univ-fhb.edu.ci

**Keywords:** anti-HIV-1 and HIV-2 antibodies, blood donors, chemiluminescent immunoassay, p24 antigen, rapid test, screening, typing

## Abstract

**Introduction:**

HIV screening by chemiluminescent immunoassay (CLIA) enables the simultaneous detection of anti–human immunodeficiency virus (HIV)‐1/2 antibodies (Abs) and the p24 antigen (Ag), ensuring high sensitivity and specificity. Viral typing is essential for the clinical follow‐up of seropositive blood donors. This study aimed to identify anti‐HIV Ab profiles among blood donors who tested positive by CLIA and to assess the concordance between CLIA screening and supplemental rapid testing.

**Methods:**

A descriptive study was conducted at the Regional Blood Transfusion Center of Bouaké from November 2024 to February 2026. Blood donors were screened for HIV using two CLIA platforms (MAGLUMI and MACCURA). The testing algorithm consisted of CLIA screening, followed by HIV Bioline rapid testing for serological typing and a follow‐up test on a new blood sample. CLIA‐reactive samples were classified as concordant reactive when confirmed by the HIV Bioline test and as presumed false positives when the initial CLIA‐reactive result was not confirmed by HIV Bioline and became non‐reactive on repeat CLIA testing on a new sample.

**Results:**

Among 21,643 blood donors screened, 79 (0.37%) were reactive by CLIA. A male predominance was observed (sex ratio: 2.8). The mean age was 33.9 years in men and 31 years in women. Of these, 44/79 (55.7%) were classified as concordant reactive, 19/79 (24.1%) as presumed false positives, and 16/79 (20.2%) as indeterminate (CLIA‐reactive but HIV Bioline‐negative pending follow‐up). Among the confirmed reactive samples, 34 (43.0%) were infected with HIV‐1, 1 (1.3%) with HIV‐2, and 9 (11.4%) had HIV‐1/HIV‐2 dual infection. Two cases of hepatitis B co‐infection were also identified. The MAGLUMI assay showed a specificity of 99.93%, a proportion of concordant reactive results of 77.5%, and a presumed false‐positive rate of 0.07%, whereas the MACCURA assay showed a specificity of 99.87%, a concordance of 65%, and a presumed false‐positive rate of 0.13%. Signal‐to‐cutoff (S/CO) ratios were higher in concordant reactive samples (1.89–500) than in presumed false‐positive samples (1.03–11.56).

**Conclusion:**

Despite their high performance, CLIA assays require confirmatory testing to determine the HIV type (HIV‐1 or HIV‐2), reduce false‐positive results, and ensure a reliable diagnosis.

## 1. Introduction

The human immunodeficiency virus/acquired immunodeficiency syndrome (HIV/AIDS) epidemic continues to be a public health concern despite the development of prevention and treatment strategies [1]. Côte d’Ivoire, with an estimated HIV prevalence of 1.82%, is among the West African countries most affected by the HIV pandemic [2]. Infected individuals may live for years without exhibiting specific symptoms [3], which poses a significant risk of accepting potentially infected blood donations in transfusion centers. Screening, which is mandatory for blood donations, remains the only means of detecting infection.

HIV exists in two main types. HIV‐1, the first‐identified type, is the most virulent form and accounts for the majority of AIDS cases worldwide. HIV‐2, although sharing several biological characteristics with HIV‐1, is distinguished by lower pathogenicity, certain antigenic differences, and a more limited geographic distribution [4].

West Africa has the highest global prevalence of HIV‐2 infection, accounting for a substantial proportion of HIV infections, estimated at 10%–20% in some populations [5]. Although HIV‐2 is less transmissible than HIV‐1, the absence of specific antiretroviral therapy leads in most cases to progression to AIDS. However, disease progression is slower than in HIV‐1 infection, with a more gradual decline in CD4+ T‐cell counts [6]. In addition, HIV‐2 is intrinsically resistant to non‐nucleoside reverse transcriptase inhibitors (NNRTIs) and certain protease inhibitors. Consequently, misclassification of HIV‐2 as HIV‐1 or failure to identify HIV‐1/HIV‐2 co‐infections may result in inappropriate treatment regimens and therapeutic failure [7].

Over the past decade, chemiluminescent immunoassays (CLIAs) have become the reference technology for the serological screening of transfusion‐transmissible infections because of their excellent analytical sensitivity, specificity, and ability to detect both antibodies (Abs) and antigens (Ags). Fully automated CLIA analyzers improve laboratory efficiency by providing standardized, high‐throughput testing with minimal operator intervention, thereby reducing the turnaround time and analytical variability [8]. Several commercial platforms, including the MAGLUMI X3 (Snibe) and the MACCURA i1000 (MACCURA Biotechnology), are currently used for routine screening of blood donors for HIV, hepatitis B virus (HBV), and hepatitis C virus (HCV). These systems have been progressively implemented in blood transfusion services across sub‐Saharan Africa, including Côte d’Ivoire, to strengthen blood safety and meet the increasing demand for reliable large‐scale donor screening.

HIV screening by CLIA enables the simultaneous detection of Ab and the HIV‐1 p24 Ag, providing high sensitivity and specificity, even at low concentrations. This method allows for early, accurate, and reliable detection, reducing the serological window period to approximately 2 weeks [9], thereby improving transfusion safety. However, despite the high accuracy of these fourth‐generation assays, false‐positive results may occur [10, 11], potentially leading to negative psychological and social impacts for the affected donors.

HIV serotyping (HIV‐1 or HIV‐2 infection) is therefore essential for confirming positive results and ensuring appropriate clinical follow‐up of seropositive donors. The objective of this study was to identify anti‐HIV Ab profiles among blood donors diagnosed as seropositive by a CLIA and to assess the concordance between CLIA screening and supplemental rapid testing.

## 2. Materials and Methods

This descriptive study was conducted from November 2024 to February 2026 at the Regional Blood Transfusion Center of Bouaké, located in north‐central Côte d’Ivoire. It was a matter of non‐remunerated voluntary blood donors who were recruited either at the Bouaké Regional Blood Transfusion Center or during mobile blood collection campaigns conducted in the city of Bouaké and its surrounding areas.

### 2.1. Inclusion and Exclusion Criteria

All consenting blood donors who successfully completed the blood donation were included. Individuals in whom blood donation could not be performed were excluded, particularly in cases of failed venipuncture (“dry tap”), adverse reactions such as malaise during the procedure, or a known previous diagnosis of HIV, HBV, HCV, or syphilis.

Regarding the sampling strategy, all consecutive blood donations performed during the defined study period were included, without selection or random sampling, provided that they met the inclusion criteria. Sociodemographic data (age, sex, and number of previous donations) were collected using donor questionnaires and securely stored in the national blood bank information system.

### 2.2. Patient Samples

It involved serum samples collected from blood donors between November 2024 and October 2025. A total of 21,643 serum samples were collected and screened for HIV.

Samples were analyzed using the MAGLUMI X3 CLIA system (Snibe Diagnostic) and the MACCURA i1000 CLIA assay for HIV screening. Blood donors found to be reactive by chemiluminescence were subsequently serotyped using the Bioline HIV‐1/2 3.0 assay (Abbott).

Blood samples were collected in serum tubes containing a clot activator. After collection, serum samples were stored at 4°C for 24–48 h. For pre‐analytical processing, tubes were centrifuged at 4000 × *g* for 5 min to allow the separation of serum from the erythrocyte clot. A single CLIA test required 200 μL of sample, whereas the Bioline assay required 10 μL.

HIV‐reactive samples were also tested for HBV and HCV using the MAGLUMI X3 CLIA system (Snibe Diagnostic) and the MACCURA i1000 CLIA system. The markers assessed included hepatitis B surface Ag (HBsAg), anti‐HCV Abs, as well as the HIV Ag and anti‐HIV Abs.

The number of previous donations made by each reactive donor was recorded, and their ABO blood group was determined. All assays were performed and interpreted strictly in accordance with the manufacturers’ instructions.

### 2.3. Principle of HIV CLIA

Initial screening of blood donations was performed using two CLIA analyzers, MAGLUMI and MACCURA. The analyzers were used sequentially during the study period according to reagent availability within the laboratory. Therefore, the analyzer used for each donation depended solely on reagent availability at the time of screening and not on donor characteristics. No participant selection or allocation was made based on demographic or clinical criteria.

The MAGLUMI X3 system (Snibe Diagnostic) and the MACCURA i1000 analyzer are fully automated CLIA platforms designed for high‐throughput testing in clinical laboratories. They provide a broad range of immunological assays, including tests for infectious diseases, such as HIV.

The assay is based on the principle of CLIA, in which specific Ag or Abs present in the patient’s blood sample react with the corresponding labeled reagents. The resulting chemiluminescent signal was measured by the automated analyzer to determine the presence or absence of HIV markers.

The HIV Ag/Ab CLIA assay is a qualitative CLIA designed for the simultaneous detection of HIV‐1 p24 Ag and Abs to HIV‐1 and/or HIV‐2 in human serum or plasma. The assay follows a two‐step sandwich format.

In the first step, paramagnetic microparticles coated with monoclonal anti‐HIV p24 Abs, together with microparticles coated with HIV‐1‐ and HIV‐2–specific Ags, are added to the reaction cuvette containing the donor’s sample and mixed.

In the second step, acridinium ester–labeled monoclonal anti‐HIV p24 Abs, as well as acridinium ester–labeled HIV‐1 and HIV‐2 Ags, are added to the reaction vessel. These reagents form a sandwich complex with either the HIV‐1 p24 Ag or anti‐HIV‐1/HIV‐2 Abs present in the donor’s sample that have been captured by the microparticles, respectively.

The chemiluminescent substrate was added, and its reaction with the acridinium ester–labeled immune complex formed on the microparticles triggers the chemiluminescent reaction. The resulting chemiluminescence is measured in relative light units (RLU) using a photomultiplier.

The RLUs generated during the reaction are proportional to the amount of HIV‐1 p24 Ag and/or anti‐HIV‐1 or anti‐HIV‐2 Abs present in the sample. The presence or absence of HIV‐1 p24 Ag and/or anti‐HIV‐1 or HIV‐2 Abs in the samples is determined by comparing the chemiluminescent signal to the cutoff value established during calibration. Samples with an signal‐to‐cutoff (S/CO) ratio < 1.00 are considered non‐reactive for HIV, while those with an S/CO ≥ 1 are considered reactive.

### 2.4. Principle of Anti‐HIV‐1/HIV‐2 Ab Detection by Bioline Test

The Bioline HIV‐1/2 test is a third‐generation rapid qualitative assay for the detection of Abs specific to HIV‐1 and HIV‐2 in the serum.

The Bioline HIV‐1/2 3.0 test uses a membrane strip pre‐coated with recombinant HIV‐1 capture Ags (gp41 and p24) in test region 1 and recombinant HIV‐2 capture Ag (gp36) in test region 2. The sample, together with colloidal gold–conjugated recombinant HIV Ags (gp41, p24, and gp36), migrates along the membrane by capillary action toward the test regions (T). A visible line appears when an Ag–Ab–Ag gold complex forms, allowing highly sensitive and specific detection.

A colored control line (C) appears in the result window to indicate that the test has functioned correctly. Test regions 1 and 2 in the result window correspond to the outcomes for HIV‐1 and HIV‐2 Abs, respectively.

The simultaneous presence of test line 1 and control line C indicates a positive result for anti‐HIV‐1 Abs.

The simultaneous presence of test line 2 and control line C indicates a positive result for anti‐HIV‐2 Abs.

The simultaneous presence of test lines 1 and 2 along with control line C indicates a positive result for anti‐HIV‐1 and/or anti‐HIV‐2 Abs.

### 2.5. Follow‐Up Monitoring

Donors whose samples tested reactive for HIV were followed up for repeat testing after a minimum of 2 months. HIV screening was repeated using the MAGLUMI X3 (Snibe Diagnostic) and MACCURA i1000 CLIA assays, and anti‐HIV Ab serotyping was reassessed using the Bioline HIV‐1/2 3.0 assay (Abbott).

### 2.6. Diagnostic Algorithm

**Figure 1:**
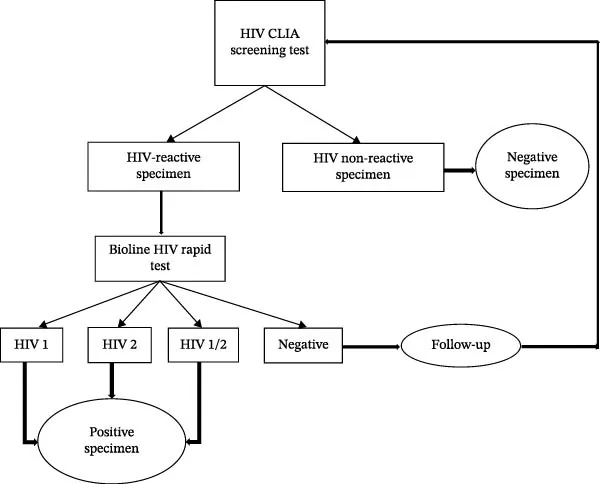
Schematic diagram of the diagnostic algorithm.

Reactive CLIA samples underwent HIV Bioline serological typing, followed by repeat CLIA testing on a newly collected sample after 2 months. Nucleic acid testing (NAT) and Western blot were not performed because these assays were not routinely available at the Regional Blood Transfusion Center of Bouaké during the study period owing to technical and resource constraints. Consequently, the diagnostic algorithm used in this study relied exclusively on serological testing (Figure 1).

### 2.7. Variables and Operational Definitions

A “concordant reactive” was defined as any specimen reactive by the CLIA test and positive by the Bioline test.

A “presumed false‐reactive” was defined as any specimen initially reactive by CLIA but negative by the Bioline test, which became non‐reactive on CLIA at follow‐up testing performed at least 2 months later on a new blood sample.

An “indeterminate” result was defined as any specimen reactive by CLIA but negative by the Bioline test.

### 2.8. Statistical Analysis

Categorical and continuous variables were described using frequencies, means, and standard deviations, respectively. The agreement rate between CLIA screening results and supplemental rapid testing, as well as specificity, was calculated using Microsoft Excel.

Missing data were completed, when possible, by contacting donors via telephone in order to minimize incomplete information.

### 2.9. Informed Consent

Written informed consent was obtained from all study participants, allowing the publication of the results with full respect for anonymity.

### 2.10. Ethical Approval Statement

The project entitled “Anti‐HIV serological profiles and concordance of chemiluminescent immunoassay screening with a supplemental rapid test among blood donors in Côte d’Ivoire” received ethical approval from the Scientific Ethics Committee (CSE) of the National Blood Transfusion Center of Côte d’Ivoire (CNTSCI). Approval No. 05‐24/CNTSCI/CSE/Db, signed in Abidjan on September 05, 2024.

## 3. Results

Among the 21,643 blood donors tested by CLIA, 79 (0.37%) were reported as reactive during screening, with signal‐to‐cutoff ratios ranging from 1.01 to 500. Among the reactive donors, there was a male predominance compared to females (sex ratio: 2.8). The age of male donors ranged from 19 to 57 years, with a mean of 33.9 years, whereas female donors ranged from 18 to 52 years, with a mean of 31 years. The distribution of ABO blood groups among reactive donors was: O (47.2%), A (16.7%), B (31.9%), and AB (4.2%). The number of donations made by the 79 HIV‐reactive donors ranged from 1 to 15 (Table 1).

**Table 1:** Sociodemographic characteristics of blood donors.

Characteristics	Values
Age (years), mean ± SD	33.1 ± 11.5
Male sex, *n* (%)	58 (73.4%)
Female sex, *n* (%)	21 (26.6%)
Number of donations, median (IQR)	01 (01–05)

Of the total 21,643 donors screened by CLIA, 6097 were tested using the MACCURA automated system, while 15,546 were tested using the MAGLUMI automated system.

**Figure 2:**
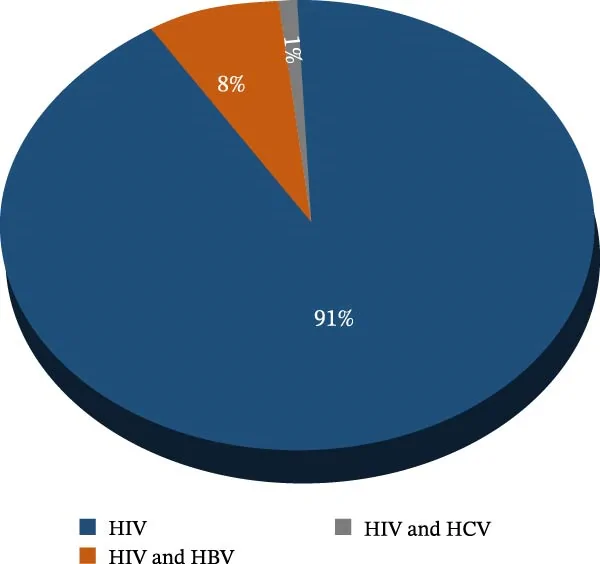
HIV coinfection with HBV and/or HCV.

Among the 79 reactive donors, 28 were identified as reactive using the MACCURA automated system, with signal‐to‐cutoff ratios ranging from 1.03 to 157.4, while 51 were identified using the MAGLUMI automated system, with ratios ranging from 1.01 to 500. Seven donors presented with co‐infections, including six (8%) with hepatitis B and one (1%) with hepatitis C (Figure 2).

The analysis of anti‐HIV Ab profiles among the 79 donors initially reactive by CLIA revealed the presence of anti‐HIV Abs in 43 individuals (54.4%) confirmed as positive by the Bioline serotyping assay (Table 2).

**Table 2:** Prevalence of HIV serotypes after initial screening.

Serotype	Male		Female		Total	
	Number	(%)	Number	(%)	Number	(%)
HIV‐1	20	25.3	13	16.5	33	41.8
HIV‐2	1	1.3	0	0	1	1.3
HIV‐1 and HIV‐2	4	5.1	5	6.3	9	11.4
Negative	32	40.5	4	5.1	36	45.6

The signal‐to‐cutoff ratios among donors in whom the HIV serotype was identified ranged from 16.4 to 500. Two donors serotyped as HIV‐1 presented a co‐infection with HBV. Among these 43 donors positive for HIV‐1 and/or HIV‐2, 13 were tested using the MACCURA analyzer and 30 using the MAGLUMI analyzer.

In contrast, for the 36 donors whose viral serotype was negative at screening, the ratios ranged from 1.01 to 95.1. Five of these donors had co‐infections, including four positive for hepatitis B and one with anti‐HCV Abs. The ratio values are summarized in Table 3.

**Table 3:** Signal‐to‐cutoff (S/CO) ratios among HIV‐serotyped donors after initial screening.

Serotype	Maglumi		Maccura	
	Number	Ratio	Number	Ratio
HIV‐1	22	[22.5–500]	11	[19.9–157.4]
HIV‐2	1	104	0	—
HIV‐1 and HIV‐2	7	[70.7–500]	2	[16.4–28.7]
Négative	21	[1.01–42.4]	15	[1.03–95.2]

Among the 79 donors initially reactive by the CLIA assay, 33 (41.8%) returned for follow‐up testing at least 2 months after the initial screening. Of these donors, 21 belonged to the group of 36 donors who were initially CLIA‐reactive but were not confirmed by the Bioline HIV serotyping test.

Among these 21 donors, 19 tested negative by the CLIA assay on a new follow‐up sample, confirming that their initial CLIA‐reactive results were presumed to be false positives. Of these 19 donors, 11 had initially been tested using the MAGLUMI analyzer and 8 using the MACCURA analyzer.

The remaining two donors among the 21 who underwent follow‐up testing remained CLIA‐reactive. In one donor, the Bioline HIV serotyping assay remained negative despite persistent CLIA reactivity. In contrast, the other donor, who initially showed low CLIA reactivity (ratio = 1.89) with a negative HIV serotyping result, subsequently developed a confirmed HIV‐1 infection at the first follow‐up visit, performed 7 months after the initial screening, with a marked increase in the CLIA ratio (247).

Following the follow‐up assessment, the number of indeterminate results decreased to nine specimens for MAGLUMI and seven for MACCURA.

Overall, 44 samples were classified as concordant reactive cases: 43 were identified at the initial screening and one additional case confirmed during follow‐up. Of these 44 samples, 31 were initially screened using the MAGLUMI analyzer and 13 using the MACCURA analyzer.

Among CLIA‐reactive samples, 77.5% of MAGLUMI‐reactive samples with ratios ranging from 22.5 to 500 were also reactive by the supplemental rapid test, whereas 65% of MACCURA‐reactive samples with ratios ranging from 16.4 to 157.4 were reactive by the supplemental rapid test.

The proportion of concordant reactive results was calculated using the following formula:

Concordance %=(Concordant reactive)Concordant reactive + Indeterminate×100.

The MAGLUMI HIV Ag/Ab assay demonstrated a specificity of 99.93% (15,495/15,506) for HIV diagnosis. Similarly, the MACCURA HIV Ag/Ab assay showed a specificity of 99.87% (6069/6077) for HIV detection.

The specificity was calculated using the following formula:

Specificity (%)=Negatives specimenNegatives specimen +Presumed false positives×100.

• Number of negative specimen = total number of samples − number of initially reactive samples.

Among the 79 donors who initially tested reactive for HIV, the presumed false‐positive rate was 0.07% (11/15,546) for the MAGLUMI assay and 0.13% (8/6097) for the MACCURA assay. Donors with presumed false‐positive results had a mean age of 35.7 years (range 19–57), whereas concordant reactive donors had a mean age of 34.7 years (range 18–56). Seventeen out of 19 (89.5%) presumed false‐positive donors were male. Among the presumed false‐positive cases, one donor had a hepatitis B infection. Signal‐to‐cutoff ratios for presumed false‐positive samples ranged from 1.03 to 11.56.

The number of previous donations ranged from 1 to 3 among donors with an identified HIV serotype. In contrast, among donors whose follow‐up testing confirmed an initial presumed false‐positive result, the number of previous donations ranged from 1 to 15; of these, three were first‐time donors.

## 4. Discussion

The HIV prevalence identified in this study was low and consistent with the 0.38% prevalence previously reported in a study based on a smaller sample size [12]. It was also lower than the global HIV prevalence estimated at 0.7% by the World Health Organization (WHO) [13].

By comparison, substantially higher prevalence rates have been documented across several African settings, including 1.84% in the study by Tagnya et al. [14], 3.8% in Ghana [15], 10.6% in Nigeria [16], and 16.7% in Ethiopia [17].

This difference may be attributed to increased public awareness and the implementation of multiple programs aimed at effective control of viral infections, which have been a major public health concern, particularly HIV/AIDS, over the past decade. However, the HIV prevalence among our donors remains relatively high compared to other studies that reported prevalences of 0.1% [18, 19].

The study population consisted predominantly of young adults, with a mean age comparable to that reported in most studies on blood donation [12, 18].

The male predominance among blood donors observed in our study has also been reported in several African studies, with males accounting for more than 80% of donors in most cases [20–24].

The HIV Ag/Ab assays based on CLIA technology, including those developed by MAGLUMI and MACCURA, are qualitative screening tests recognized for their high analytical reliability and diagnostic performance. Over the past decades, CLIA technology has undergone substantial development and is now widely employed in the diagnosis of infectious diseases, particularly HIV infection and hepatitis B and C viruses [25–27].

Cases of HIV–HBV and HIV–HCV co‐infection have been reported in several studies. In Mali, 38 cases of HIV–HBV co‐infection and 9 cases of HIV–HCV co‐infection were documented [28]. Similarly, Gendler and Pascuccio [29] reported 2 cases of HIV–HBV co‐infection and 21 cases of HIV–HCV co‐infection. In contrast, Habibou et al. [12] identified only a single case of HIV–HBV co‐infection.

Consistent with the findings of Parker et al. [30] and Habibou et al. [12] HIV‐1 was the predominant viral type identified in our study. However, in contrast to these studies, the co‐circulation of HIV‐1 and HIV‐2 was observed, with HIV‐1/HIV‐2 dual infection detected in 11.4% of confirmed HIV‐positive donors.

In our study, the MAGLUMI assay demonstrated a proportion of concordant reactive results of 77.5% across signal‐to‐cutoff ratio values ranging from 22.5 to 500, whereas the Maccura assay showed a concordance of 65% for ratios between 16.4 and 157.4.

The proportion of concordant reactive results remains incomplete owing to the loss to follow‐up of some participants at the time of control testing. The absence of these final data may have introduced a selection bias.

Furthermore, NAT/PCR could not be performed to definitively confirm true‐positive, false‐positive, and false‐negative results. The use of this reference test would have enabled a more accurate assessment of the diagnostic performance of the test under evaluation, particularly its positive predictive value (PPV).

Alonso et al. [31] reported that samples tested with the Architect HIV Ag/Ab assay with S/CO > 100 were highly likely to be true positives, with a PPV of 99.3%. However, for samples with S/CO < 100, it was not possible to determine whether they were true or false positives.

Parker et al. [30] found that an S/CO >180 with the BioPlex HIV Ag‐Ab assay corresponded to a PPV of 62.9%. Kim et al. [32] reported that an S/CO of 6.6 yielded a PPV ranging from 31.2% to 89.7% but with low sensitivity. Jensen et al. [33] achieved a PPV of 100% with a sensitivity of 67.4% for samples with an S/CO >151.17.

Previous reports [32, 34] have indicated that the PPV of fourth‐generation HIV assays can be low. In our study, the specificity of the MAGLUMI HIV Ag/Ab assay (99.93%) appeared excellent and was broadly consistent with the findings reported by Wang et al. [35]. Their results showed that the MAGLUMI assay had a specificity of 99.85% (5262/5270) for samples from blood donors and hospitalized patients and a sensitivity of 100.00% (614/614) in HIV‐positive samples.

The MACCURA HIV Ag/Ab assay demonstrated a specificity of 99.87%, a value comparable to that obtained for MAGLUMI or the Architect assay (specificity 99.94%) in Wang et al.’s study [36]. These findings indicate that the specificity values observed in our study are excellent and align with those reported in other studies on fourth‐generation HIV assays [37, 38].

The sensitivity of the Maglumi HIV Ag/Ab assay enabled the detection of a donor with low‐level reactivity during the initial screening (ratio = 1.89), whereas HIV serotyping was negative at that stage. 7 months after this screening, HIV‐1 infection was confirmed, demonstrating the ability of fourth‐generation assays to detect the p24 Ag before the appearance of anti‐HIV Abs.

The work of Ly et al. [39] showed that fourth‐generation HIV p24 Ag/Ab detection assays exhibit high sensitivity following nucleic acid amplification testing while maintaining a specificity above 99%.

Indeed, after HIV‐1 infection, specific HIV‐1 markers appear in the blood in the following chronological order: HIV‐1 RNA, p24 Ag, anti‐HIV‐1 immunoglobulin M (IgM) Abs, and then anti‐HIV‐1 IgG Abs. The time between HIV acquisition and test reactivity depends on the target being detected, the point at which this target becomes detectable after infection, the concentration of the target in the sample, the sample volume analyzed, and the analytical sensitivity of the assay [4].

Although reported sensitivities and specificities of fourth‐generation HIV assays remain above 99% in many studies, cases of false‐positive results have been documented, leading to discrepancies between screening techniques and confirmatory methods [37, 40–43].

In our study, the presumed false‐positive rate of the MAGLUMI assay is lower than the false‐positive rate reported by Zhang et al. [44] for fourth‐generation tests (0.08%) and by Kim et al. [32] (0.22%). Conversely, the presumed false‐positive rate of the MACCURA assay was higher than the false‐positive rate reported by Zhang et al. [44] but lower than that reported by Kim et al. [32].

The S/CO ratios of presumed false‐positive samples ranged from 1.03 to 11.56, whereas those of concordant reactive samples ranged from 1.89 to 500. For comparison, Kim et al. [32] reported false‐positive ratios ranging from 1.00 to 34.59 and true‐positive ratios ranging from 7.28 to 739.98. Parker et al. [30] reported false‐positive ratios ranging from 1 to 4.

These data indicate that false‐positive results are generally associated with low S/CO ratios. This observation has also been noted by other authors, who reported that the false‐positive rate decreases as the assay ratio increases [36, 45, 46].

For the Architect HIV Ag/Ab Combo assay, Wang et al. [36] reported that the false‐positive rate was 100% for samples with a ratio between 1 and 10 but decreased to 0% when the ratio was ≥30.

The majority of presumed false‐positive results were observed among male donors aged between 19 and 57 years. However, Zhang et al. [44] reported a significantly higher rate of false‐positive results among female donors, as well as among donors younger than 18 years and older than 65 years.

Several factors have been associated with the occurrence of false‐positive results in serological testing, including certain infections, such as viral hepatitis and malaria, which can cause cross‐reactivity with HIV Ags, as well as autoimmune diseases and pregnancy [47–50].

Given that Côte d’Ivoire is a malaria‐endemic country, malaria constitutes a significant potential confounding factor due to the cross‐reactivity it may induce with HIV serological assays. However, the malaria status of blood donors was not assessed in this study, precluding evaluation of its possible contribution to the false‐positive results observed.

Among the presumed false‐positive HIV cases observed in our study, one donor had a HBV infection. Consistently, Wang et al. [36] reported that five false‐positive HIV screening cases were associated with HBV infection.

However, this study has an important limitation. Among blood donors who initially tested HIV‐reactive, only 41.8% returned for the follow‐up assessment performed 2 months after the initial screening. The main reasons reported were that donors had relocated from the study area or could no longer be contacted using the contact information they had provided. This substantial loss to follow‐up may have affected the estimation of the PPV as well as the proportion of false‐positive results observed in our study.

Beyond these logistical constraints, it is also possible that some donors deliberately avoided confirmatory testing. Receiving an initial HIV‐reactive result may trigger considerable emotional distress, including denial, fear of a confirmed diagnosis, and anxiety about its social consequences. In many sub‐Saharan African settings, despite remarkable advances in HIV care and treatment, including widespread access to antiretroviral therapy and substantial improvements in life expectancy, HIV‐related stigma remains pervasive. The fear of discrimination, social rejection, and the psychological burden associated with a potential HIV diagnosis may discourage individuals from returning for confirmatory testing [51]. Therefore, the observed loss to follow‐up likely reflects a combination of logistical and psychosocial factors, limiting our ability to ascertain the true HIV status of all initially reactive donors and potentially influencing the study estimates of PPV and false‐positive rates.

## 5. Limitations

This study has several important limitations. First, no NAT/PCR was performed to definitively resolve discordant CLIA and Bioline results. Consequently, it was not possible to determine whether samples with low S/CO ratios represented early acute HIV infections during the serological window period or true false‐positive results. Second, more than half of the initially HIV‐reactive blood donors did not return for follow‐up testing, resulting in a substantial loss of follow‐up. This limitation may have introduced selection bias and affected the accuracy of the estimated PPV and the proportion of false‐positive results. Finally, given that Côte d’Ivoire is a malaria‐endemic country, assessing the malaria status of blood donors during the study would have allowed us to evaluate the potential contribution of malaria to the false‐positive results observed.

## 6. Conclusion

This study aimed to identify anti‐HIV Abs among blood donors who were reactive by CLIA. A substantial discrepancy was observed between CLIA results, particularly for low S/CO ratios, and HIV serotyping results. The CLIA method demonstrated high sensitivity for the detection of p24 Ag and/or Abs against HIV‐1 and HIV‐2, enabling the early screening of HIV‐infected donors. However, this technique may generate false‐positive results mainly due to non‐specific immune reactions. Low S/CO ratios should therefore raise a strong suspicion of false‐positive results and require targeted confirmatory testing strategies. Although CLIA‐based screening requires confirmation by specific assays, algorithms relying solely on serological follow‐up may be vulnerable to donor loss to follow‐up.

**Nomenclature**
HIV: Human immunodeficiency virus
AIDS: Acquired immunodeficiency syndrome
HBV: Hepatitis B virus
HBsAg: Hepatitis B surface antigen
HCV: Hepatitis C virus
CLIA: Chemiluminescence immunoassays.

## Author Contributions

**M’boh Epi Reine Elisabeth N’Gou**: conceptualization, data curation, formal analysis, investigation, project administration, writing – original draft, writing – review and editing, resources. **Mamadou Yassongui Sekongo**: resources, supervision, writing – review and editing. **Saydou Kabore**: project administration, supervision, writing – review and editing. **Nardianhio Soro**: data curation, investigation. **Natacha Fleur Nenelou:** data curation, supervision, validation, writing – review and editing. **Liliane Siransy**: methodology, project administration, supervision, validation, writing – review and editing. **Bamory Dembele:** project administration, supervision, writing – review and editing.

## Funding

No funding was received for this manuscript.

## Disclosure

All authors agree to be accountable for all aspects of the work.

## Conflicts of Interest

The authors declare no conflicts of interest.

## Data Availability

The data that support the findings of this study are available from the corresponding author upon reasonable request.
